# Acute Laryngeal Dyspnea as the First Presentation of Mantle Cell Lymphoma: A Case Report and Review of the Literature

**DOI:** 10.1155/2020/8818270

**Published:** 2020-09-09

**Authors:** Mounir Ababou, Hicham El Maaroufi, Adnane Hammani, Selim Jennane, El Mehdi Mahtat, Mohammed Mikdame, Kamal Doghmi

**Affiliations:** ^1^Department of Clinical Hematology, Military Hospital Mohammed V, Mohammed V University, Rabat, Morocco; ^2^Faculty of Medicine and Pharmacy of Rabat, Mohammed V University, Rabat, Morocco

## Abstract

**Introduction:**

Acute laryngeal dyspnea is a life-threatening emergency, and the causes in adults are most often laryngeal tumors or inflammatory edema. Lymphoma of the larynx and especially the mantle cell type is extremely rare. *Case Presentation*. We report a case of a 43-year-old woman with no particular pathological history. She presented with progressive dyspnea which has evolved towards an inspiratory bradypnea that worsened until she ultimately required an emergency tracheotomy. Biopsies revealed mantle cell lymphoma. The patient has been staged IVB MIPI 6, and she was treated by immunochemotherapy followed by ASCT. The therapeutic evaluation shows a complete remission, 18 months after, and the patient was always disease free.

**Conclusion:**

The laryngeal localization of the mantle cell lymphoma is extremely rare; it may present catastrophically with acute airway obstruction. The diagnosis is mostly histological, hence the interest of deep biopsy. Given its rarity, the therapeutic strategy must be discussed case by case in a multidisciplinary consultation meeting.

## 1. Introduction

Acute laryngeal dyspnea is a life-threatening emergency, and the causes in adults are most often laryngeal tumors or inflammatory edema [1]. Laryngeal lymphoma is extremely rare, accounting for less than 1% of laryngeal tumors [2]. Less than 100 cases have been reported in the literature, including some cases of laryngeal mantle cell lymphoma [3–5]. Because of its rarity, we want to show to the medical community the clinical presentation of mantle cell lymphoma of the larynx which can be an emergency while discussing possible therapeutic choices by reviewing the literature.

## 2. Case Presentation

**Figure 1:**
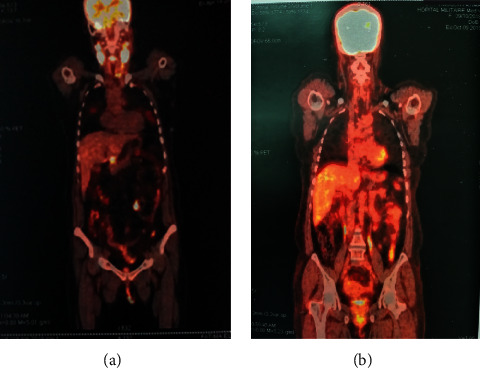
(a) PET/CT at diagnosis showed hyperpathological lymph node metabolism over and under the diaphragmatic involvement. (b) PET/CT after treatment showed a complete metabolic remission.

**Figure 2:**
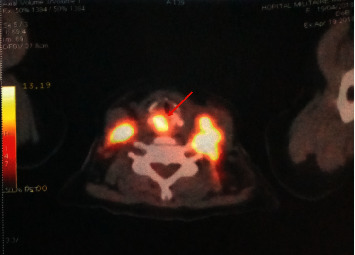
PET/CT at diagnosis showed pathological laryngeal hypermetabolism with cricoid cartilage involvement (red arrow).

We report a case of a 43-year-old woman with no particular pathological history. She presented with progressive dyspnea which has evolved towards an inspiratory bradypnea that worsened until she ultimately required an emergency tracheotomy. Laryngoscopic examination showed an obstructive mass in the right infraglottic area. A punch biopsy of larynx tumor and excision of left cervical adenopathy were performed. The anatomopathological and immunohistochemical examination revealed a blastoid variant of mantle cell lymphoma CD20+, CD5+, cycline-D1+, LCA+, BCL6+/−, MUM1+/−, CD3−, CD30−, ALK−, and Ki67 at 90%. The patient reported general signs such as weight loss and nocturnal sweats in the last six months. The ECOG (Eastern Cooperative Oncology Group) performance status was 2, and the clinical examination revealed a tumoral syndrome made of bilateral cervical lymphadenopathy. Blood test showed leukocytosis at 11300/mm^3^ with hyperlymphocytosis at 6200/mm^3^ predominantly of small mature lymphocytes and a high LDH level at 779 U/l (the upper normal level limit of the laboratory is 245U/l). The rest of the biological assessment was normal. The 18-FDG positron emission tomography (PET) scan showed an over and under diaphragmatic lymph node involvement and bilateral pathological pulmonary nodules (Figure 1(a)). A pathological laryngeal hypermetabolism with cricoid cartilage involvement (SUVmax = 15) was noticed (Figure 2), and bone marrow biopsy was negative. The patient has been staged IVB (Ann Arbor Classification), and the Mantle Cell Lymphoma International Prognostic Index (MIPI) score was 6 (high-risk group). She was treated by immunochemotherapy with 4 cycles of R-DHAOx (rituximab, high-dose aracytine, oxaliplatin, and dexamethasone), followed by autologous hematopoietic stem cell transplantation (ASCT). The therapeutic evaluation by PET scan shows a complete remission (Figure 1(b)), and it was decided to continue with maintenance therapy with rituximab every 2 months, but it was refused by the patient. At the last visit, 18 months of ASCT, the patient was always disease free.

## 3. Discussion

Acute laryngeal dyspnea is a life-threatening emergency, and the causes in adults are most often laryngeal tumors or inflammatory edema [1]. Laryngeal lymphoma in adults is uncommon, accounting for less than 1% of all cases of laryngeal malignancies. Less than 100 cases have been reported in the literature [2]. Mantle cell lymphoma (MCL) is rare and very aggressive; this subtype presents 5% of all non-Hodgkin's lymphomas [6]. Extranodal involvement in mantle cell lymphoma is common in the bone marrow, the gastrointestinal tract, and Waldeyer's ring [7]. The larynx involvement is extremely rare. In our literature review, some cases of laryngeal MCL were reported [3–5] (Table 1). Laryngeal lymphoma presents clinically in a similar fashion to squamous cell carcinoma, with symptoms such as hoarseness, dyspnea, a foreign body sensation in the throat, or stridor. Uncommonly, it may present catastrophically with acute airway obstruction requiring immediate surgical intervention as in our case [8]. Systemic symptomatology is unusual, since laryngeal lymphomas tend to remain localized for prolonged periods, although more aggressive forms tend to spread earlier [9]. There is no consensus on the treatment of MCL, and for young patients (<65 years) in good general condition, the first-line therapeutic strategy is based on the use of induction immunochemotherapy containing cytarabine followed by consolidation with intensive chemotherapy and autologous stem cell transplantation [10]. We opted for this strategy in our case. MCL is considered incurable with current therapies and has historically been associated with a poor prognosis. However, increased understanding of the disease biology has led to the development of promising novel therapies in recent years [11].

## 4. Conclusion

Mantle cell lymphoma (MCL) is a rare and very aggressive subtype of non-Hodgkin's lymphoma with poor prognosis. The laryngeal localization is extremely rare, and it may present catastrophically with acute airway obstruction. The diagnosis is mostly histological, hence the interest of deep biopsy. Given its rarity, the therapeutic strategy must be discussed case by case in a multidisciplinary consultation meeting.

## Figures and Tables

**Table 1:** Case reports of mantle cell lymphoma of the larynx.

Article	Year of publication	Age	Sex	Clinical presentation	Tracheotomy	Laryngeal localization	B Symptoms	Ann arbor stage at diagnosis	Therapy	Outcome for MCL	Status on date of publication
Our case	2020	43	F	Inspiratory bradypnea	Yes	Right infraglottic	Yes	IVB	R-DHAOX + ASCT	CR	Alive
K.L.Groom et al. [4]	2011	60	M	Progressive hoarseness	No	Left anterior false vocal cord	NA	NA	NA	NA	NA
S.Naciri [3]	2012	70	M	Progressive laryngeal respiratory distress	Yes	Subglottic	No	IIA	R-CHOP	NA	Died
Y. Ç. Kumbul et al. [5]	2019	76	M	Progressive nasal obstruction	No	Midline nasopharyngeal + left aryepiglottic fold	NA	NA	R-CHOP + radiotherapy	CR	Alive

MCL: mantle cell lymphoma; R-DHAOX: rituximab, dexamethasone, aracytine, oxaliplatin; R-CHOP: rituximab, cyclophosphamide, doxorubicine, vincristine, prednisone; CR: complete remission; NA: not available.
